# Reliability, validity and cut-off score of the Chinese version of the panic disorder severity scale self-report form in patients with panic disorder

**DOI:** 10.1186/s12888-020-02560-w

**Published:** 2020-04-15

**Authors:** Xitong Liu, Tingting Xu, Dandan Chen, Chen Yang, Pei Wang, Xiao Huang, Wenhong Cheng, Yuan Shen, Qiang Liu, Zhen Wang

**Affiliations:** 1 Shanghai Mental Health Center, Shanghai Jiao Tong University School of Medicine, No.600 South Wanping Road, Shanghai, 200030 China; 2 Department of Psychological Medicine, Zhongshan Hospital, Fudan University, No.180 Fenglin Road, Shanghai, 200032 China; 3 Department of Medical Psychology, Shanghai General Hospital, Shanghai Jiao Tong University School of Medicine, No. 100 Haining Road, Shanghai, 200080 China; 4 Department of Psychiatry, Tenth People’s Hospital of Tongji University, No.301 Middle Yanchang Road, Shanghai, 200072 China

**Keywords:** Panic disorder severity scale-self report, Reliability, Validity, Cut-off score

## Abstract

**Background:**

Panic disorder (PD) is often undiagnosed, misdiagnosed, or untreated in non-psychiatric clinical settings. Therefore, a cost-effective, accurate and easy-to-administer instrument for PD assessment is still needed. For that reason, the self-report version of the Panic Disorder Severity Scale (PDSS-SR) has been developed and suggested to be a reliable and useful tool in clinical and research settings. The current study aims to evaluate the reliability and validity of the Chinese version of the PDSS-SR and determine the cut-off score of the PDSS-SR.

**Methods:**

A total of 133 patients with PD in Shanghai were assessed by the PDSS-SR, PDSS and Hamilton Anxiety Rating Scale (HAMA). Moreover, 117 patients with non-PD anxiety and 51 healthy subjects also completed the PDSS-SR to construct a receiver operating characteristic (ROC) curve with the scores of PD patients.

**Results:**

The internal consistency (Cronbach’s α) of the PDSS-SR was 0.72–0.80, and the interrater correlation coefficient was 0.78. The results of principal component analysis and varimax rotation indicated that the PDSS-SR had a two-factor structure, with all seven items having salient loadings. The cut-off score was 4, which was associated with high sensitivity (96.03%) and specificity (61.31%).

**Conclusions:**

The findings demonstrate that these items and the total score of the PDSS-SR have acceptable reliability and validity in patients with PD and that the PDSS-SR can be used by general doctors for clinical screening in China.

## Background

Panic disorder (PD), a common psychiatric disorder, has a morbidity of 1.6–2.2% worldwide [1, 2]. A meta-analysis of the prevalence of anxiety disorders in mainland China from 2000 to 2015 showed that the pooled prevalence of PD in China for current PD is 1.08‰ (95% CI: 0.74–1.43), and the lifetime prevalence is 3.44‰ (95% CI: 2.46–4.41). In addition, compared with females, males seem to have a lower risk of developing PD (current: OR = 0.50, 95% CI: 0.32–0.77; lifetime: OR = 0.49, 95% CI: 0.33–0.72) [3]. Compared to healthy subjects, PD patients have higher unemployment rates, more significant work impairment, and a higher frequency of medical treatment and hospitalization [4], seriously affecting the normal life of the individual. Thus, more research is needed regarding the origins and treatment of PD.

The typical symptoms of PD include unexpected and recurrent panic attacks and the corresponding consequences. PD patients have an increased risk of comorbid psychosis, manic behaviour, drug abuse, depression, dysthymia and suicide. Although several effective treatments are now available, as many as half of individuals with PD are undiagnosed, misdiagnosed, or untreated [1, 5, 6], which makes it necessary to make an effort to better understand PD.

A considerable proportion of PD patients are initially diagnosed by general physicians or emergency physicians [1]. In the cardiology department, 38 to 47.1% of patients with the chief complaint of chest pain suffer from PD [7, 8]. Due to their cardiovascular and neurological symptoms, patients with PD can easily be misdiagnosed with somatic diseases, and their initial visits are often in the cardiology, emergency and neurology department [3, 9]. In Fleet’s study, 441 PD patients with chest pain as a chief complaint went to the emergency department for treatment; only 2% of subjects were diagnosed with PD [1, 10]. The low diagnosis rate seriously influences the early treatment of PD and results in heavy social and economic burdens [2, 11].

A screening tool is required for the general hospital, especially in cardiology and emergency departments, to help physicians recognize common symptoms of PD. For PD scales, such as the Panic and Agoraphobia Scale (PAS) and the Albany Panic and Phobic Questionnaire (APPQ), only the Panic Disorder Severity Scale (PDSS) and the Panic-Associated Symptom Scale (PASS) have been tested by Chinese researchers [12]. The PASS was published in 1991 when it was used to assess the core symptoms of PD in the DSM-III-R. The PDSS, a scale intended for determining severity in individuals already diagnosed with PD, is effectively utilized for the assessment, prevention, and intervention phases of PD. However, it is costly and time-consuming to train general doctors to use the PDSS [3, 13]. Hence, a cost-effective, accurate, and easy-to-administer measure for PD is still needed. For that reason, the Panic Disorder Severity Scale-Self Report (PDSS-SR) was developed as a self-reported version of the PDSS [14] to rate the overall severity of PD [5]. The PDSS-SR consists of 7 items coded on a 5-point ordinal scale (0–4), in which higher scores indicate a more severe panic attack. The PDSS-SR can be performed by patients without the help of trained physicians or interviewers. Thus, this scale is easily used for the screening of PD in general hospitals.

The cut-off score can be utilized to distinguish PD patients from non-PD patients; thus, this score may be useful as a tool to screen patients in settings such as primary care for diagnosis-related symptoms [15]. However, only one study has proposed a cut-off score worldwide, reflecting a challenge for clinical work [16]. Although many clinicians and researchers have applied the PDSS-SR in their work, there is still ambiguity regarding the diagnostic threshold of PD in China. Thus, the present study had two major aims: 1) to test the reliability and validity of the Chinese version of the PDSS-SR and 2) to determine an optimal cut-off score for the PDSS-SR.

## Methods

### Participants

A total of 133 PD patients (74 females and 58 males, one person unknown) from four hospitals (Shanghai Mental Health Center, Shanghai First People’s Hospital, Shanghai Tenth People’s Hospital and Zhongshan Hospital) were enrolled from October 2017 to March 2018. A total of 117 non-PD patients with other anxiety disorders and 51 healthy controls (HCs) were also included. All subjects aged 18–65 years provided informed consent and participated voluntarily. PD patients and non-PD patients were all diagnosed by the chief physician of psychiatry according to the ICD-10. The exclusion criteria were as follows: 1) concurrent diagnosis or past history of any other psychiatric disorder; 2) pregnancy or ≤ 6 months postpartum; 3) inability to read and understand the informed consent form or self-reported questionnaires; and 4) presentation with acute suicidality. This study was approved by the ethics committee of the Shanghai Mental Health Center.

### Measures and procedures

The PDSS-SR, a new self-report diagnostic measure of PD adapted from the PDSS, can be used by patients to monitor the severity of their symptoms in the last week. The PDSS-SR has seven items that can assess patients’ panic attack frequency, distress during panic attack, anticipatory anxiety, agoraphobic fear/avoidance, fear/avoidance of panic-related bodily sensations, work impairment and social impairment based on their rating on a five-point scale (0 = “not at all” to 4 = “most severe”). Item ratings are summed to form the total score, with higher scores indicating greater symptom severity.

The adopted PDSS is the Chinese version that has been clinically tested with good reliability and validity and better diagnostic efficiency for PD. The PDSS is a seven-item scale designed to assess the overall severity of PD symptoms by a psychiatrist or trained interviewer.

Another assessment instrument, the Hamilton Anxiety Scale (HAMA), which is a 14-item measure rated on a five-point scale (0 =“not at all” to 4 = “most severe”), is widely utilized in clinical symptom evaluation for assessing the severity of emotional and physical anxiety [17].

This study is a multicentre study with a number of patients recruited from the psychology departments of four hospitals. General demographics, disease information, PDSS and HAMA were assessed by a unified trained clinical researcher. Participants completed the self-report measures by themselves. Two weeks later, the PDSS-SR was again assessed.

### Statistical analyses

All analyses were conducted using SPSS 20.0. Internal consistency of the PDSS-SR was evaluated using Cronbach’s α. Test-retest reliability was examined using Pearson correlations between session one and session two scores (2 weeks later). Parallel validity was evaluated with Pearson correlations between the PDSS-SR (total and item scores) and the different instruments administered (PDSS and HAMA). The baseline structural validity was evaluated by exploratory factor analysis. The cut-off score was determined by performing receiver operating characteristic curve (ROC) analysis.

## Results

### Demographic properties

The mean age of the total PD sample (*n* = 133) was 37 ± 12 years. The age range in our study was 18–60 years. A total of 55.6% of the sample was female. The mean duration of the disorder (in months) was 8 (2, 24) for the whole group and the retest sample. Table 1 displays the socio-demographic properties of the different groups.

**Table 1:** Sample characteristics

		Whole sample (*N* = 133)	Test–retest sample (*N* = 74)
Age (M, SD, year)		37(12)	38(12)
Gender	Male	58	36
	Female	74	38
Education level (M,SD)		14(3)	14(3)
Illness duration (Months)		8(2, 24)	8(2, 24)
PDSS-SR score		10.53(4.56)	5.68(3.67)

Annotation: one patient default

We also compared the PD, non-PD anxiety and healthy groups according to age, sex, education and the PDSS-SR score using the Chi-square test. The results are shown below (Table 2).

**Table 2:** Chi-square test between three groups

		PD (*n* = 133)	Non-PD anxiety (*n* = 117)	HC (*n* = 51)	F	*p*
Age		37(12)	36(13)	33(8)	118.98	0.095
Gender	Male	58	49	20	0.35	0.838
	Female	74	68	31		
Education		13.83(3.19)	12.99(3.61)	14.39(2.21)	45.99	0.052
PDSS-SR		10.49(4.58)	6.58(6.11)	0.43(1.15)	201.82	0.000**

** represents *p* < 0.001, similarly hereinafter

### Reliability

#### Internal consistency reliability

Cronbach’s α of the PDSS-SR was 0.78, which indicated the high reliability of this scale, and the Cronbach’s α of each item with the sum of the remaining items (if the item was deleted) is shown in Table 2. For the whole group (*n* = 133), the items of work impairment and social impairment showed a high correlation (0.65 and 0.63, respectively), while the item of distress during panic attacks showed a low correlation (0.34).

#### Test–retest reliability

For the group of panic disorder patients who were reassessed after 2 weeks, the Pearson correlation coefficient (r^2^) between the PDSS-SR scores at baseline and after 2 weeks was 0.42, which reached statistical significance, and the correlation of each item was between 0.13 and 0.37 (Table 3).

**Table 3:** Statistical results of internal consistency

Items	Alpha if item-deleted	Corrected item-total correlation r1	Corrected item-total correlation r2
Panic attack frequency	0.76	0.45	0.22
Distress during panic attacks	0.80	0.34	0.13
Severity of anticipatory anxiety	0.75	0.52	0.37**
Agoraphobic fear/avoidance	0.75	0.53	0.34**
Panic related sensation fear/avoidance	0.75	0.52	0.29**
Work impairment	0.72	0.65	0.35**
Social impairment	0.73	0.63	0.33**

Note: ** Correlation is significant at the 0.01 level (2-tailed)

### Validity

#### Factor analysis

According to the appropriate tests, we obtained a Kaiser-Meyer-Olkin of 0.75, and the *χ*^*2*^ from Barlett’s test was 281.03 (*p* < 0.001), which was suitable for performing factor analysis. After the principal component analysis (PCA), a model with two correlated factors was constructed, with the first of the three items (i.e., the symptoms of panic attack) loading on the second factor (panic attacks factor, focuses on physical symptoms), and the other four on the first factor. These two factors with eigenvalues > 1 could explain 60.59% of the total variance. The rotated component matrix is shown in Table 4.

**Table 4:** Rotated component matrix

Items	Factor 1	Factor 2
Panic attack frequency	0.18	0.74
Distress during panic attacks	0.02	0.76
Severity of anticipatory anxiety	0.46	0.49
Agoraphobic fear/avoidance	0.81	0.08
Panic related sensation fear/ avoidance	0.85	0.02
Work impairment	0.65	0.46
Social impairment	0.67	0.41

#### Parallel validity

Regarding the correlations of measures from baseline, the Pearson correlation coefficient, r, for the PDSS-SR total score was 0.68 with the PDSS and 0.42 with the HAMA, and they were both statistically significant (*p* < 0.001).

### Cut-off score

**Fig. 1:**
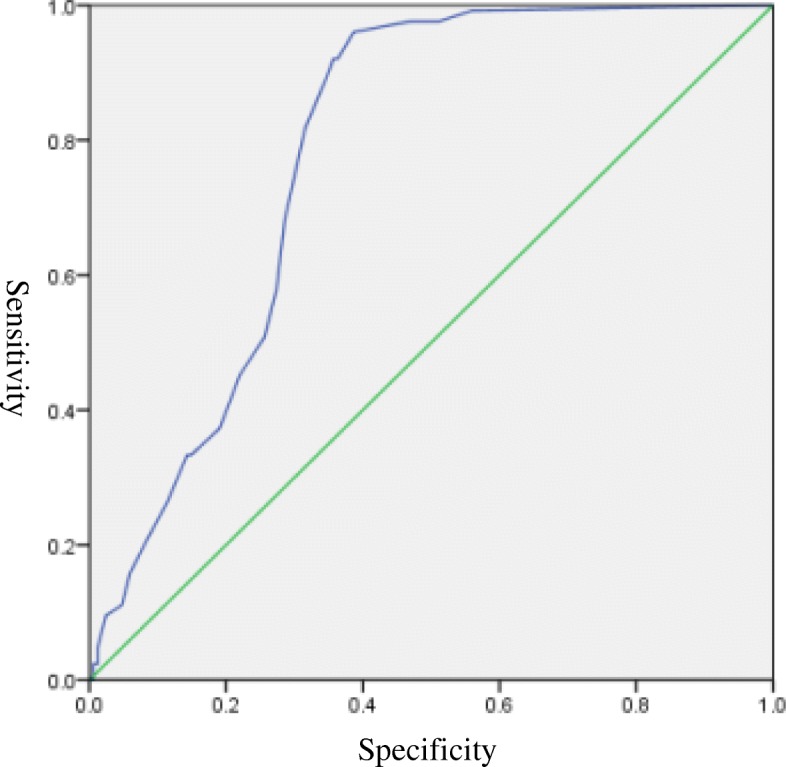
Receiver-operating characteristic curve (ROC) analysis of the PDSS-SR

A total of 117 patients without panic disorders but with other anxiety disorders and 51 healthy subjects were tested using the PDSS-SR, and ROC curves were plotted with data from patients with PD. When the Jordan index calculated from the curve coordinates was 0.5734, we obtained the optimal sensitivity (96.03%) and specificity (61.31%), and it can be determined that the corresponding demarcation is divided into 4 points. The area under the curve (AUC) was 0.782 (S.E. = 0.03, *p* < 0.001), indicating that the scale has certain diagnostic accuracy (Fig. 1).

## Discussion

Panic disorder is a kind of anxiety disorder with autonomic nervous dysfunction as the main symptom. Patients often go to various general hospitals for physical examination instead of mental health centres in the first stage. This study aimed to assess the reliability and validity of the Chinese version of the PDSS-SR to show that it can be widely used as an ideal tool for outpatients in general hospitals. We also determined the cut-off score to improve the diagnostic effect of the scale. The results demonstrate that these items and the total score of the PDSS-SR had acceptable reliability and validity over a two-week period in patients with PD, which is similar to the findings of a previous multicentre study [12]. The cut-off score was 4, which was associated with high sensitivity and ideal specificity.

We found that when the cut-off was 4, the AUC was 0.783, showing that the effectiveness of this model in detecting PD was acceptable. The PDSS-SR was found to have a high sensitivity of 96.03%, which meant that people with PD only had a 3.97% chance of being missed, and most patients could be identified and diagnosed. However, the specificity was 61.31%, indicating that the false positive rate for this score was 38.69%, which meant there was a 38.69% chance that patients will be misdiagnosed. Compared with the previous study in which the cut-off was 8.5 (with 89% sensitivity and 100% specificity) [16], our research obtained a lower specificity based on the 4-point cut-off, which may be influenced by cultural differences between the East and the West. Nevertheless, we restricted the PD participants and excluded patients with comorbidities and obtained a higher sensitivity, which is more important for screening scales, especially for nonpsychiatric departments in general hospitals. Patients with suspected panic disorder may be referred to psychiatric departments for further diagnosis and treatment, where false positives are partially offset.

The test–retest reliability was 0.42, and the correlations of each item were between 0.13 and 0.37. The reliability coefficient of the retest was lower than those obtained in previous studies [12, 13], whose intervals were quite short (1–5 days) after the initial test. The retest interval of this study was 2 weeks, during which the characteristics measured by the scale may have changed. Within 2 weeks, most patients were treated with SSRIs; thus, improvement in symptoms may have affected this indicator. In conclusion, the low retest reliability does not indicate poor PDSS-SR reliability.

We used factor analysis to determine construct validity and separated out 2 factors. The interpretable variance was 60.59%. Items 1–3 loaded on the first factor, and the other 4 items loaded on the second factor. Although the result was inconsistent with the previous one-factor model of PDSS-SR [13], it was consistent with the two-factor structure of PDSS and the general features of panic disorder, to be specific, cognitive components (i.e., expectation anxiety, phobic features), physical components and anxiety, even if the entries under the factor were different from those of previous studies [18, 19].

Although many psychometric properties of the PDSS-SR were reported in detail and the cut-off value of panic disorder was calculated, some limitations should also be considered. First, the specificity was lower than that in a previous study, which would lead to increased misdiagnosis and, to some extent, increase the cost of medical treatment for people. Second, we did not consistently control the patients’ treatment during the 2-week interval. Although most patients were treated with SSRIs, some patients received no treatment, which may have affected the retest reliability.

## Conclusion

In conclusion, our study calculated a new and reasonable cut-off for the Chinese version of the PDSS-SR and confirmed that it is a tool with acceptable reliability and validity over a two-week period in patients with PD, demonstrating its convenience for the clinical screening of PD, especially in general hospitals.

## Data Availability

The datasets used and/or analysed during the current study are available from the corresponding author on reasonable request.

## Abbreviations

PD: Panic disorder
PDSS: Panic Disorder Severity Scale
PDSS-SR: Panic Disorder Severity Scale Self-Report
HC: Healthy control
HAMA: Hamilton Anxiety Scale
ROC: Receiver-operating characteristic curve
PCA: Principal component analysis
AUC: Area under the curve
